# SCN1A polymorphisms influence the antiepileptic drugs responsiveness in Jordanian epileptic patients

**DOI:** 10.5937/jomb0-34544

**Published:** 2023-03-15

**Authors:** Rami Abduljabbar, Tamimi Duaa Eid, Al-Motassem Yousef, Saeed Ramzi Mukred, Mohammed Zawiah

**Affiliations:** 1 University of Jordan, School of Pharmacy, Department of Biopharmaceutics and Clinical Pharmacy, Amman, Jordan; 2 University of Jordan, School of Medicine, Department of Pharmacology, Amman, Jordan; 3 University of Al Hodeidah, College of Clinical Pharmacy, Department of Pharmacy Practice, Al Hodeidah, Yemen

**Keywords:** epilepsy, drug resistance, antiepileptic drugs, SCN1A polymorphisms, epilepsija, rezistencija na lekove, antiepileptički lekovi, SCN1A polimorfizmi

## Abstract

**Background:**

The aim of this study was to evaluate whether the voltage-gated sodium channel alpha subunit 1 (SCN1A) gene polymorphisms influence the responsiveness of Jordanian epileptic patients to antiepileptic drugs (AEDs).

**Methods:**

A total of 72 AEDs-treated epileptics were polymerase chain reaction (PCR)-genotyped for six single nucleotide polymorphisms (SNPs), including SCN1A rs2298771, rs3812718, rs3812719, rs2217199, rs2195144 and rs1972445. Genotype and allele distributions in drug-responsive and drug-resistant patients were compared. The six SNPs haplotypes were examined, and the linkage disequilibrium (LD) was assessed.

**Results:**

The genotypes of drug-resistant and drug-responsive groups were in Hardy-Weinberg equilibrium. Three genetic polymorphisms of the SCN1A gene seemed to influence the resistance to AEDs, on the level of alleles and genotypes. Data revealed that rs2298771 G allele, rs3812719 C allele, and rs2195144 T allele increased the risk of developing AEDs-resistance (OR=2.9; 95%CI= 1.4-5.9, p=0.003; OR=2.4; 95%CI=1.2-4.7, p=0.01; OR=2.3; 95%CI=1.2-4.7, p=0.01), respectively. Haplo type analysis of SCN1A polymorphisms revealed high-degree LD associated with resistance to AEDs. A synergetic effect appears with highly significant association in GCCATG haplotype of rs2298771, rs3812718, rs3812719, rs2217199, rs2195144, and rs1972445 respectively (OR=2.8; 95%CI=1.5-6.2, p=0.002).

**Conclusions:**

Data suggests that SCN1A polymorphisms could influence the resistance to AEDs in Jordanian epileptics at three SNPs (rs2298771; rs3812719; rs2195144). Additionally, haplotype analysis indicated a substantial degree of LD between the six SCN1A polymorphisms. Further investigation with larger sample size is needed to confirm the results of the current study.

## Abstract (sr)

**Uvod:**

Cilj ove studije bio je da se proceni da li polimorfizmi gena alfa podjedinice 1 natrijumovog kanala (SCN1A) utiču na reakciju na antiepileptičke lekove (AED) kod epileptičnih pacijenata u Jordanu.

**Metode:**

Ukupno 72 pacijenta sa epilepsijom koji su tretirani sa AED su bili genotipizovani polimerazom lančane reakcije (PCR) za šest pojedinačnih nukleotidnih polimorfizama (SNP), uključujući SCN1A rs2298771, rs3812718, rs3812719, rs2217199, rs2195144 i rs1972445. Poređene su distribucije genotipa i alela kod pacijenata koji reaguju na lekove i pacijenata koji su rezistentni na lekove. Ispitano je šest haplotipova SNP-a i procenjena je neravnoteža veze (LD).

**Rezultati:**

Genotipovi grupa otpornih na lekove i grupa koje reaguju na lekove su bili u Hardy-Weinberg-ovoj ravnoteži. Činilo se da tri genetska polimorfizma gena SCN1A utiču na otpornost na AED, na nivou alela i genotipova. Podaci su otkrili da alel rs2298771 G, alel rs3812719 C i alel rs2195144 T povećavaju rizik od razvoja rezistencije na AED (OR=2,9; 95%CI=1,4-5,9, p=0,003; OR=2,4; 95%CI= 95%CI=1,2-4,7, p=0,01; OR=2,3; 95% CI=1,2-4,7, p=0,01), respektivno. Analiza haplotipa polimorfizama SCN1A otkrila je LD visokog stepena povezanu sa otpornošću na AED. Sinergetski efekat se pojavljuje sa visoko značajnom asocijacijom u GCCATG haplotipu rs2298771, rs3812718, rs3812719, rs2217199, rs2195144 i rs1972445, respektivno (OR=2,8; 95%CI=1,5-6,2, p=0,002).

**Zaključak:**

Podaci ukazuju da bi polimorfizmi SCN1A mogli da utiču na otpornost na AED kod pacijenata iz Jordana sa epilepsijom kod tri SNP (rs2298771, rs3812719, rs2195144). Pored toga, analiza haplotipa je ukazala na značajan stepen LD između šest SCN1A polimorfizama. Potrebno je dalje istraživanje na većem uzorku da bi se potvrdili rezultati trenutne studije.

## Introduction

Epilepsy is a neurological disease characterized by an enduring predisposition to generate epileptic seizures due to abnormal neuronal activity in the brain [1]. Epilepsy is one of the oldest and most common diseases that affect individuals of any age and ethnicity, affecting approximately 1–2% of the population [2]. Most patients with epilepsy respond to antiepileptic drugs (AEDs). However, about one-third of epileptic patients develop repeated seizures, despite the efficacy of treatment at the optimal dose regimen. They are then considered resistant to antiepileptic therapy [3]. Despite efforts to predict the AEDs responsiveness, the mechanisms underlying the resistance to AEDs in epilepsy treatment are still not well-understood [4].

The voltage-gated sodium channel alpha subunit 1 (*SCN1A*) gene is located on chromosome 2q24 and contains 27 exons [5]. The *SCN1A* encodes the Nav1.1 protein, a molecular target for sodium channel blockers-antiepileptic drugs (SCB-AEDs) [6]. The sodium channel protein undergoes voltage-dependent changes in conformation that regulate conductance through the channel pore. At resting membrane potentials, most channels are in closed states. In the state of response to membrane depolarization, channels activate within a few hundred microseconds, resulting in flux of sodium through the open channel pore, and then convert to non-conducting inactivated states within a few milliseconds [7]. These ion channels are molecular targets for many AEDs [8], which block ionic conductance through these channels [9] [10]. The SCB-AEDs have been a cornerstone in treating focal and generalized tonic-clonic seizures for more than 70 years. The sodium channels reveal a large pore-forming alpha subunit associated with two smaller beta subunits [11] [12]. Evidence suggests that the channel blocking AEDs do so mainly by binding the alpha subunit [10].

Genetic variation at different sites in *SCN1A* contributes to a wide range of seizure types. Different mutations in *SCN1A *gene have been identified to cause monogenic clinical phenotypes of epilepsy in addition to more common nonmonogenic epilepsies [5] [13] [14] [15]. The importance of *SCN1A *lies not only in its possible causal role in epilepsy but also in its potential influence on the efficacy of AEDs [16].

To clarify the above association, we performed this study to explore further the relationship between *SCN1A *polymorphisms (rs2298771, rs3812718, rs3812719, rs2217199, rs2195144, and rs1972445) and the responsiveness to SCB-AEDs among Jordanian patients with epilepsy.

## Materials and methods

### Subjects

The study approached 100 patients who had been diagnosed and prescribed AEDs for one year or more at the Neurology clinic in Al-Basheer Hospital in Jordan. The recruitment duration was over 3 month's period (June-September 2018). Human subjects' confidentiality and rights were maintained throughout the study. The study was approved by the School of Pharmacy Scientific Research committee, Deanship of Academic Research, and the Institutional Review Board (IRB) of Al-Basheer Hospital (MBA/IRB/8147 on May 27th, 2018). All recruited subjects provided written informed consent. 72 patients met the inclusion criteria of the study and were enrolled. Patients with any diagnosed genetic abnormality or who were non-compliant with their AEDs were excluded. Patients with pathologies that may increase the occurrence of epileptic seizures were also excluded from this study, including imaging abnormalities such as neurological or systemic degenerative disorders, tumour, atrophic lesions, tuberculoma, and multiple neurocysticercoses [17] [18] [19], gross neurological deficiencies (mental retardation, motor/speech), hematopoietic [20], traumatic, metabolic, and psychiatric disturbances [21], and cancers.

Data were obtained from medical records or during patients' interviews. For each patient, the following clinical information was recorded: age, gender, clinical history of the patient, his/her family history of epilepsy, lifestyle, and date of starting treatment with AEDs. Recruited patients were classified as AED-responsive and AED-resistant. Positive family history was defined as the incidence of epilepsy in immediate relatives. Drug resistance was defined as the occurrence of at least four seizures for 1 year during treatment [22].

### Genotyping and sequencing

Blood samples were collected from 72 patients taking SCB-AEDs. Genomic DNA was extracted by the standard salting-out extraction method [23]. Polymerase Chain Reaction (PCR) followed by sequencing for six *SCN1A *SNPs: rs2298771, rs3812718, rs3812719, rs2217199, rs2195144, and rs1972445 was done. Appropriate forward and reverse primer sets were prepared (Table 1). PCR conditions were: 2 μL (50–100 ng) of DNA was amplified by adding 10 μL of OneTaq® Quick-Load® master mix (NEB, UK), the reverse, and forward primers, 1.5 μL of each (10 μmol/L), and 3.5 μL of water for injection. The thermal cycler program was as follows: an initial denaturation step at 94 °C for 3 min followed by 39 cycles of denaturation at 94 °C for 30 seconds, annealing for 15 seconds at 62 °C, an extension step at 68 °C for 60 seconds, and a final extension step at 68 °C for 5 min. PCR products were visualized on agarose gel (2%) stained using RedSafe®. Sharp PCR products were good candidates for sequencing. The Sanger sequencing technique was used in our study by GENEWIZ Technical Support Group, USA (http://www.genewiz.com).

**Table 1:** Primers sequences for SCN1A SNPs Homo sapiens chromosome 2, GRCh38.p12 Primary Assembly Sequence ID: NC_000002.12.

SNP rs#	Primer Sequence	Position
rs3812718, rs3812719, rs2217199,<br> rs2195144 and rs1972445	Forward (F): 5 -TCACATGATGGGTCCGTCTC -3’	166052811-166052830
	Reverse (R): 5 - GCAGCCCAACAACACTTACC -3’	166053813-166053794
rs2298771	F: 5 - GTCACAGTAAGACTGGGGTTGT - 3’	166036136-166036157
	R: 5 - CAACCTTGCAGCCACTGATG - 3’	166036483-166036464

### Data management and statistical analysis

Data were analyzed using Statistical Package for the Social Sciences (SPSS) software version 22 (SPSS® Inc, Chicago, USA). Chi-square or Fisher exact tests were used to compare demographic and clinical characteristics between the drug-resistant and drug-responsive groups. Allele and genotype frequencies among recruited patients were estimated from the sequencing results and read by Chromas Lite software version 2.1.1. Frequencies of genotypes and alleles were analyzed in concordance with the Hardy-Weinberg equilibrium. Associations between genotype, haplotype, and drug-response were tested by Chi-square or Fisher exact test. The strength of associations was estimated by odds ratios (OR) and its 95% confidence interval (95% CI). Haplotypes were identified by the Haploveiw™ 4.2 Software [14]. All tests were two-tailed, and *p*<0.05 was considered statistically significant.

## Results

### Participants' description and clinical characteristics

Of the 72 patients with epilepsy taking SCB-AEDs, 30 were classified as drug-responsive, and 42 were drug-resistant. Males and females were almost equally represented in the study; 55.6 % (N=40) and 44.4 % (N=32), respectively. At the time of the interviews, the patient's average age was 31.1 (±13) years, whereas their average age at onset of seizure was 19.9 (±13) years.

Patients were more likely to be prescribed one AED (N=29, 40.3%) or two AEDs (N=32, 44.4%). Fewer patients received three AEDs or more (N=11, 15.3%). Most recruited patients were with unknown aetiology (N=42, 58.3%), while 30 patients (41.7%) were with known aetiology. Most patients had negative family history for epilepsy (N=63, 87.5%) (Table 2). Trauma is the most common identified cause of epilepsy (N=14, 19.4%).

**Table 2:** Comparison of clinical and patient characteristics according to resistance to AEDs Values expressed as N and %; N: number; % was calculated out of available data. Fisher’s exact test or chi-square test (as appropriate) were used to estimate p-value. OR: odds ratio. CI: confidence interval. AEDs: Antiepileptic Drugs. OR and its 95% CI was not calculated for p>0.05. *: p-value was calculated for the three groups together using chi-square test.

Categories	Sub-categories	Resistant patients<br>(N=42)		Responsive patients<br>(N=30)		OR (95 % CI)	p-value
		N	%	N	%		
Gender	Male	28	70	12	30	3 (1.1–7.9)	0.02
	Female	14	43.8	18	56.2		
Age at interview	>29.5 years	29	76.3	9	23.7	5.2 (1.9–14.4)	0.002
	≤29.5 years	13	38.2	21	55.3		
Age at seizure onset	≤17.5 years	19	51.4	18	48.6		0.3
	>17.5 years	23	65.7	12	34.3		
Family history	Yes	3	33.3	6	66.7		0.1
	No	39	61.9	24	38.1		
Number of AEDs	Mono-therapy	10	34.5	19	65.5	0.18 (0.06–0.5)	0.001*
	Bi-therapy	22	68.8	10	31.2	2.2 (0.8–5.8)	
	3 AEDs	10	90.9	1	9.1	9.06 (1.09–75.2)	
AEDs prescribed	Carbamazepine	29	69	13	31	1.2 (0.5–2.8)	0.02
	Topiramate	2	28.6	5	71.4		0.1
	Lamotrigine	12	85.7	2	14.3	3.5 (0.7–16.4)	0.01
	Valproate	28	63.6	16	36.4		0.1

Comparisons of the demographic and clinical characteristics between drug-resistant and drug-responsive subjects are shown in Table 2. Females were significantly less frequent in the resistant group than in the drug-responsive group (OR=0.33; 95%CI=[0.1–0.9); *p*=0.02). Further subclassification of patients based on median age revealed that patients older than 29.5 years were significantly more prevalent in the resistant group than in The prevalence of resistance to AEDs was significantly higher in patients treated with more than one AED than those with monotherapy (OR=5.5; 95%CI=[4.3–7.2); *p*=0.001). Epileptic patients with three AEDs or more were 9.06 times more likely to be observed in the resistant group than in the responsive group (OR=9.06; 95%CI=[1.09–75.2), *p*=0.001). Patients taking carbamazepine were more likely to be observed in the resistant group than those not taking carbamazepine (OR=1.2; 95%CI=[0.5–2.8); *p*=0.02). Also, patients taking lamotrigine were more frequent in the resistant group than those not taking lamotrigine (OR=3.2; 95%CI=[00.7–14.9); *p *= 0.01).

### Allelic and genotypic association of SCN1A polymorphisms with AEDs responsiveness

Table 3 shows the distribution of genotypes and allele frequencies of the aforementioned *SCN1A *SNPs. Our genotyping results for all polymorphisms were consistent with the Hardy-Weinberg equilibrium (p>0.05). We found a significant association between rs2298771, rs3812719, and rs2195144 polymorphisms and AED responsiveness. For rs2298771, results showed that the drug-resistant patients were significantly more likely to have GG genotype than the AA or AG genotypes than the drug-responsive patients (OR=10.3; 95%CI=[1.2– 84.8); *p*=0.01). Epileptic patients with G alleles were 2.9 times more likely to be observed in the resistant group compared to those with A alleles (OR=2.9; 95%CI=[1.4–5.9), *p*=0.003).

**Table 3:** Allele and genotype frequencies of SCN1A polymorphisms Fisher’s exact or Chi-square (as appropriate) were used to estimate the p-value, p: Probability. p-value of <0.05 is considered significant. OR: odds ratio. CI: confidence interval. *: The p-value was calculated for the three groups using the Chi-square test. MAF: minor allele frequency. H-W freq.: Hardy-Weinberg frequency.

SNP rs#	Genotype	Total<br> patients<br> (N=72)		Resistant<br> patients<br> (N=42)		Responsive<br> patients<br> (N=30)		OR	95% CI	p-value	MAF	Expected<br> genotype<br> (H-W freq.)<br> N (%)
		N	%	N	%	N	%					
rs2298771	AA	22	30.6	8	19	14	46.7	0.3	0.1–0.8	0.007*	0.43	23.4 (32.5)
	AG	38	52.7	23	54.8	15	50	1.7	0.7–4.2			35.3 (49.0)
	GG	12	16.7	11	26.2	1	3.3	10.3	1.2–84.8			13.3 (18.5)
	AG+GG	50	69.4	34	81	16	53.3	3.7	1.3–10.7	0.02		
	AA	22	30.6	8	19	14	46.7	0.3	0.1–0.8			
	AA+AG	60	83.3	31	73.8	29	96.7	0.1	0.01–0.8	0.01		
	GG	12	16.7	11	26.2	1	3.3	10.3	1.2–84.8			
	A allele	82	56.9	39	46.4	43	71.7	0.3	0.2–0.7	0.003		
	G allele	62	43.1	45	53.6	17	28.3	2.9	1.4–5.9			
rs3812718	TC	38	52.8	21	50	17	56. 7			0.2*	0.47	35.9 (49.9)
	CC	19	26.4	14	33.3	5	16. 7					20.1 (27.9
	TT	15	20.8	7	16.7	8	26. 7					16.0 (22.2)
	TC+TT	53	73.6	28	66. 7	25	83. 3			0.1		
	CC	19	26.4	14	33.3	5	16. 7					
	TC+CC	57	79.2	35	83.3	22	73. 3			0.3		
	TT	15	20.8	7	16. 7	8	26. 7					
	T allele	68	47.2	35	41.7	33	55			0.1		
	C allele	76	52.7	49	58.3	27	45					
rs3812719	CA	39	54.1	24	57.1	15	50	1.3	0.5–3.4	0.03*	0.44	35.4 (49.2)
	CC	12	16.7	10	23.8	2	6. 7	4.4	0.9–21.7			13.8 (19.2)
	AA	21	29.2	8	19.0	13	43. 3	0.3	0.1–0.9			22.8 (31.6)
	CA+AA	60	83.3	32	76.2	28	93. 3	0.2	0.05–1.1	0.05		
	CC	12	16.7	10	23.8	2	6. 7	4.4	0.9– 21.7			
	CA+CC	51	70.8	34	81	17	56. 7	3.3	1.1–9.3	0.02		
	AA	21	29.2	8	19.0	13	43. 3	0.3	0.1–0.9			
	C allele	63	43.8	44	52.4	19	31.7	2.4	1.2–4.7	0.01		
	A allele	81	56.2	40	47.6	41	68.3	0.4	0.2–0.8			
rs2217199	GA	38	52.8	21	50	17	56. 7			0.2*	0.47	35.9 (49.9)
	AA	19	26.4	14	33.3	5	16. 7					20.1 (27.9
	GG	15	20.8	7	16. 7	8	26. 7					16.0 (22.2)
	GA+GG	53	73.6	28	66. 7	25	83. 3			0.1		
	AA	19	26.4	14	33.3	5	16. 7					
	GA+AA	57	79.2	35	83.3	22	73. 3			0.3		
	GG	15	20.8	7	16. 7	8	26. 7					
	G allele	68	47.2	35	41.7	33	55			0.1		
	A allele	76	52.8	49	58.3	27	45					
rs2195144	CT	39	54.1	24	57.1	15	50	1.3	0.5–3.4	0.03*	0.44	35.4 (49.2)
	TT	12	16.7	10	23.8	2	6. 7	4.4	0.9–21.7			13.8 (19.2)
	CC	21	29.2	8	19.0	13	43. 3	0.4	0.1–1.3			22.8 (31.6)
	CT+CC	60	83.3	32	76.2	28	93. 3	0.2	0.05–1.1	0.05		
	TT	12	16.7	10	23.8	2	6. 7	4.4	0.9–21.7			
	CT+TT	51	70.8	34	81	17	56. 7	3.3	1.1–9.3	0.025		
	CC	21	29.2	8	19.0	13	43. 3	0.3	0.1–0.9			
	C allele	81	56.2	40	47.6	41	68.3	0.4	0.2–0.8	0.01		
	T allele	63	43.8	44	52.4	19	31.7	2.3	1.2–4.7			
rs1972445	AG	38	52.8	21	50	17	56. 7			0.2*	0.47	35.9 (49.9)
	GG	19	26.4	14	33.3	5	16. 7					20.1 (27.9
	AA	15	20.8	7	16. 7	8	26. 7					16.0 (22.2)
	AG+AA	53	73.2	28	66.7	24	82.8			0.1		
	GG	19	26.8	14	33.3	5	17.2					
	AG+GG	57	78.9	35	83.3	21	72.4			0.2		
	AA	15	21.1	7	16.7	8	27.6					
	A allele	68	47.2	35	41.7	33	55			0.1		
	G allele	76	52.8	49	58.3	27	45					

In addition, CC genotype at rs3812719 was significantly more frequent in resistant patients when compared with the responsive patients (OR=4.4; 95%CI=[0.9–21.7); *p*=0.03). The patients with C alleles were 2.4 times more likely to be observed in the non-responsive group compared to those with A alleles (OR=2.4; 95%CI=[1.2–4.7), *p*=0.01). Moreover, the TT genotype patients at rs2195144 were 3 times more likely to develop resistance (OR=4.4; 95%CI=[0.9–21.7), *p*=0.05), and the patients carrying T alleles were 2.3 times more likely to be observed in the resistant group compared to those with C alleles (OR=2.3; 95%CI=[1.2–4.7),* p*=0.01). However, there was no significant association between rs3812718, rs2217199, and rs1972445 polymorphisms and AEDs responsiveness.

### Haplotype association of SCN1A polymorphisms with AEDs responsiveness

**Figure 1:**
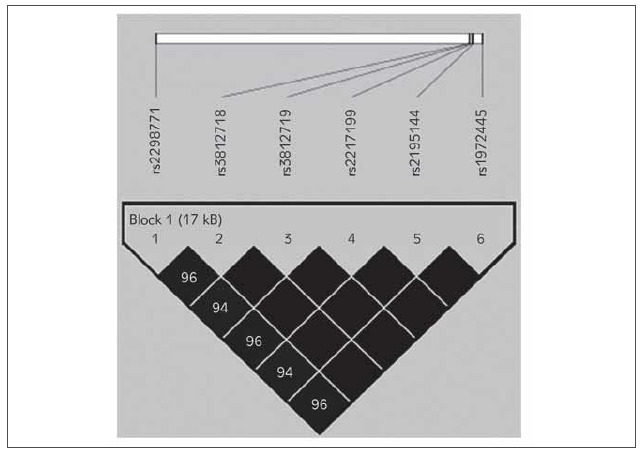
Linkage Disequilibrium (LD) plot among the six SNPs of SCN1a. Standard LD colour scheme (D´/LOD) key: Low D´ and low LOD white; Low D´ and high LOD white; High D´ and low LOD grey; High D´ and high LOD black. Values in each square represent 100*|D´|.

Haplotypic analysis indicated a strong general degree of linkage disequilibrium (LD) of *SCN1A *polymorphisms. Figure 1 graphically depicts the strength of LD. There was strong LD between the six SNPs (rs2298771, rs3812718, rs3812719, rs2217199, rs2195144, and rs1972445) that were associated with resistance to AEDs (Figure 1). A synergetic effect appears with highly significant association in GCCATG haplotype of rs2298771, rs3812718, rs3812719, rs2217199, rs2195144, and rs1972445 respectively (OR=2.8; 95%CI=[1.8–6.7), p=0.002). The *SCN1A *haplotypes that are significantly associated with non-responsive to AEDs were summarized in Table 4.

**Table 4:** Haplotype association of SCN1A polymorphisms with response to AEDs Fisher’s exact or Chi-square (as appropriate) were used to estimate the p-value, p: Probability. OR: odds ratio. CI; confidence interval. Significant p-value (< 0.05), OR and its 95% CI was not calculated for p>0.05.

SNPs	Haplotype	Total patients<br> (N=72)<br>(#of chromosomes)		Resistant patients<br> (N=42)<br> (#of chromosomes)		Responsive patients<br> (N=30)<br> (#of chromosomes)		OR	95%CI	*p*-value
		N	%	N	%	N	%			
(rs2298771,<br> rs3812718,<br> rs3812719,<br> rs2217199,<br> rs2195144,<br> rs1972445)	ATAGCA	66.8	46.4	34	40.5	32.8	54.6			0.0931
	GCCATG	80	41.6	44	52.3	16	26.6	2.8	1.5–5.6	0.002
	ACAACG	12.2	8.5	5	5.9	7.2	12			0.1957
	ACCATG	3	2.1	0	0	3	5	0.2	0.019–2.2	0.04
	GTAGCA	1.2	0.9	1	1.2	0.2	0.4			0.6
	GCAACG	0.8	0.6	0	0	0.8	1.3			0.3

## Discussion

Resistance to AEDs is one of the biggest challenges of epilepsy care. Inter-individual variations in AEDs responsiveness between patients receiving the same therapy can be attributed to the genetic profile. The *SCN1A *gene, highly polymorphic and commonly distributed in the brain, plays an essential role in one of the regulated sodium channels. The *SCN1A *genetic polymorphisms can impact the effectiveness of SCB-AEDs by altering the pharmacological goal sensitivity [16]. Consequently, detecting *SCN1A *genetic polymorphisms may lead to early recognition of patients at high risk of resistance to SCB-AEDs and an individualized antiepileptic therapy strategy [24]. In Jordan, there are previous studies conducted among the Jordanian epileptic population, but with genes other than SCN1A. Tamimi et al. [25] reported no association between ABCB1 SNPs and resistance to AEDs on the level of alleles nor on the level of genotypes but revealed significant associations at the level of haplotypes.

The epileptic patients were classified as either drug-responsive or drug-resistant. This classification was based on the definition utilized by Sterjev et al. [22] who considered resistance to AEDs as the occurrence of at least four seizures over 12 months through treatment It is important to remember that the prevalence of AEDs resistance is highly affected by the used definition of resistance that changes widely among different studies. All definitions were summarized in the 2015 meta-analysis [26]. Accordingly, in the current group of patients, »AEDs resistance« varied dramatically based on definitions from as small as 13.8% to as high as 70.8%. Utilizing Kim et al. [27] definition which defines AEDs resistance as »the occurrence of at least three seizures at the maximum tolerated doses over one year before recruitment with trials of more than three effective antiepileptic drugs,« AEDs resistance would be only 13.8%. However, the figure rises to 70.8% of the samples when using other definitions like the International League Against Epilepsy (ILAE) definition, which states that patients are considered to be non-responsive when at least two AEDs accurately administered for at least one year at full adequate doses, and epileptic seizures continued [28]. Among these two extremes, it has been decided to utilize the Sterjev et al. [22] definition to define resistance to AEDs in this study, amounting to 58% of participants.

Our observations suggested the significant involvement of *SCN1A *SNPs (rs2298771, rs3812719, and rs2195144) in modulating responsiveness to AEDs. Interestingly, to date, no previous studies have reported the effect of *SCN1A *SNPs (rs3812719 and rs2195144) on the responsiveness to AEDs. Further, extensive studies on different ethnicities are needed to confirm our findings. With regards to rs2298771 polymorphism, which is a common exonic SNP in the *SCN1A *gene, the GG genotype and G alleles were more likely to develop resistance to AEDs. Up to date, several studies investigated the role of this SNP in resistance to AEDs and have yielded conflicting results. El Fotoh et al. [29] reported that the AG genotype and G allele frequency was significantly higher in AED-resistant patients than in AED-responsive patients. A meta-analysis conducted by Yi Bao et al. [24] also confirmed this association through four studies (1 Indian, 2 Han Chinese, 1 Italian) in which the A allele was significantly correlated with responsiveness to SCB-AEDs, while the decreased risk of the resistance was observed for AA genotype carriers. The Indian study [30] revealed that patients with AA genotype were more likely to be responsive to SCB-AEDs. In addition, the Han Chinese study [6] found that the seizure-free rate in the A allele was significantly higher than that in the G allele and the AA genotype carriers were 59.9%.

On the other hand, an earlier meta-analysis of four Asian studies (3 Malaysian, 1 from Hong Kong) [31] revealed that neither alleles nor genotypes were significantly associated with drug responsiveness in epilepsy. Additionally, a recent Kosovar study showed no significant association with carbamazepine metabolism [32]. Apparently, conflicting results could be attributed to the differences in ethnicity [20] [33] and definitions of drug responsiveness [33]. On the other hand, our current study showed no association among SCN1A SNPs (rs3812718, rs1972445, and rs2217199). Regarding rs3812718, there were conflicting results from different studies. A previous meta-analysis by Haerian and colleagues [31] reported that none of the examined studies (1 Japanese, 1 Spanish, 1 Italian, 1 Indian, 3 Malaysian, and 1 from Hong Kong) showed a significant association with lack of response to AEDs in SCN1A rs3812718 polymorphism. Moreover, an Austrian study conducted in 2008 did not find a significant difference in the average carbamazepine doses between the genotype groups [34].

Alternatively, a more recent Japanese study demonstrated an association between the AA genotype and carbamazepine-associated epilepsy and confirmed the frequency of the AA genotype was higher in AED-resistant epilepsy than in AED-responsive epilepsy. However, the AA genotype was associated with a 2.7-fold increase in the risk for carbamazepineresistant epilepsy [35]. On the other hand, the maximum and maintenance dose of carbamazepine and phenytoin by AA and AG carriers in rs3812718 in British, Chinese, and Greek cohorts of patients [36] [37] [38]. The reason for the discrepancy between the present findings and those of previous studies may be due to the use of different AEDs and dosing strategies. Regarding rs1972445, only one previous study [39] investigated the effect of this SNP on response to multiple AEDs and was consistent with the current study. On the other hand, regarding rs2217199, no previous published studies have investigated its association with responsiveness to AEDs. Further investigations among different ethnicities are needed for the confirmation of any association.

Haplotype analysis showed a strong association between AEDs drug resistance and *SCN1A *haplotypes containing the six SNPs. A synergetic effect appears with a highly significant association in GCCATG haplotype of rs2298771, rs3812718, rs3812719, rs2217199, rs2195144, and rs1972445, respectively. Our results confirmed previous findings that demonstrated the significant association of *SCN1A *haplotypes composed of *SCN1A *rs2298771 and rs3812718 with maintenance dosages of carbamazepine [40]. Comparing haplotype pattern distributions revealed that patients with AA and AG haplotypes composed of *SCN1A *rs3812718 and rs2298771 were more likely to require higher maintenance dosages. On the other hand, a previous study investigated the association of AED responsiveness with *SCN1A *haplotypes [10]. They studied 8 SNPs from *SCN1A*, two of which are similar to our study (rs2298771 and rs3812718). The *SCN1A *haplotypes were not associated with resistance to AEDs. The discrepancy with the present findings may be because of the selected SNPs, differences in the sample size, ethnicity, and AEDs. To date, no previous have been published studies about the association between *SCN1A *haplotypes for the six SNPs and resistance to AEDs.

*SCN1A *is a large gene (139 Kbp), with many exons. It would be difficult to take a direct, resequencing approach to *SCN1A *in a genetic association study [16]. Unfortunately, there is little prior information concerning its pattern of LD. Hence, the selection of the minimum number of SNPs needed for a genetic association study is not known and is mostly population-dependent [16]. The current haplotype analysis revealed a strong degree of LD between the studied six *SCN1A *polymorphisms. In essence, two sets of SNPs had complete LD, (rs3812718, rs2217199, rs1972445) and (rs3812719, rs2195144). Hence, knowledge of three SNPs will suffice (rs2298771, rs3812718, rs3812719).

Three SNPs could be used in an association study with only modest or no reduction in power of that study. These important findings should be further confirmed in a larger study with more initial SNPs included. A limitation to current findings is to pinpoint the causative SNP. The LD across the entire block is so high that a causative SNP could lie anywhere in the block. Functional assays will be required in order to assess which of the many putative causal variants are the important ones.

Although our study has the strength of considering new polymorphisms in *SCN1A *genes that influence the responsiveness to AEDs, it also has some limitations: (a) the sample size is small, making it inappropriate to make subgroup analyses (b) our study was done among Jordanian population which may explain the inconsistent results. Further large studies from different populations are required to confirm our findings and to draw results from different comparisons.

In conclusion, our study provides valuable information that suggests the association of *SCN1A *genetic polymorphisms with resistance to AEDs. Additionally, *SCN1A *haplotypes influence resistance to AEDs. Results showed that tagged SNPs identified in one population may not be the same in others. It has been suggested that the most promising approach in genetic association studies is to select the smallest number of tagging SNPs for analysis in clinical trials; 4 SNPs are more predictable for the *SCN1A *gene. We recommend conducting a prospective cohort study with more epileptic patients to confirm current findings. It is recommended to adopt a block design based on gender, age, and number of AEDs, rather than utilizing a convenience sample to overcome the unequal distribution of confounders.

## Dodatak

### Statements and declarations

#### Ethics approval and consent to participate

The study was approved by the School of Pharmacy Scientific Research committee, Deanship of Academic Research, and the Institutional Review Board (IRB) of Al-Basheer Hospital (MBA/IRB/8147 on May 27^th^, 2018). All recruited subjects provided written informed consent.

#### Consent for publication

All authors have seen and approved the manuscript for submission.

#### Availability of data and materials

The data sets generated during the study are available from the corresponding author on reasonable request.

#### Acknowledgement

The authors would like to express their appreciation to the physicians and staff nurses of the neurology department in Al-Basheer Hospital for their huge assistance.

#### Funding information

This study was funded by the dean of scientific research, the University of Jordan, grant no (2017-2018/119).

#### Author contributions

AY: Supervision, Conceptualization, Methodology, Software, Formal analysis, Writing – review & editing. RA: Conceptualization, Investigation, Data curation, Methodology, Software, Formal analysis, Writing – original draft. DT: Investigation, Methodology, Software, Formal analysis, Writing – original draft. RMS: Conceptualization, Investigation, Data curation, Methodology. MZ: Investigation, Writing – review & editing.

### Conflict of interest statement

All the authors declare that they have no conflict of interest in this work.
